# Metabolic and morphometric analysis of allometric and total liver growth in Post-Hatch chickens

**DOI:** 10.1007/s11306-025-02250-2

**Published:** 2025-04-12

**Authors:** Heidi Van Every, Carl J. Schmidt

**Affiliations:** 1 Center for Bioinformatics and Computational Biology, University of Delaware, Newark, DE USA; 2 Department of Animal and Food Sciences, University of Delaware, Newark, DE USA; 3 GlaxoSmithKline, Collegeville, PA USA

**Keywords:** Chicken, Liver, Metabolome, Maturation, Allometry, Growth

## Abstract

**Introduction:**

: This study examines metabolic and morphometric changes in chicken liver metabolism during the post-hatch growth period (days 4–20). During this period, liver metabolism transitions from using yolk-derived lipids to feed derived carbohydrates and proteins. The period also encompasses distinct growth phases with implications for metabolic impacts on total and allometric (proportional) growth.

**Objectives:**

Identify shifts in metabolites and pathways that occur during the change in nutrients and relate these to patterns of total and allometric liver growth.

**Methods:**

Liver samples were collected every other day between days 4–20 and analyzed using metabolomic and morphometric approaches to relate metabolic changes to growth. Principal component analysis (PCA) and orthogonal partial least squares discriminant analysis (OPLS-DA) were used to identify trends in the data. Cross-validation ANOVA, and network analyses were applied to evaluate metabolic changes across the time periods.

**Results:**

Three liver growth periods were defined. Period A (days 4–8) exploited stored nutrients to support rapid growth. Period B (days 10–14) was transitional as stored nutrients were depleted and feed became the major metabolic driver. By period C (days 16–20) the liver is fully dependent on feed. Positive allometric growth occurs predominantly during period A while the organ continues to grow throughout the entire time.

**Conclusions:**

Metabolic pathways exhibit distinct networks as nutrient resources change over the early post-hatch period. These findings provide a framework for understanding how nutrient-driven metabolism influences liver scaling and functional maturation.

**Supplementary Information:**

The online version contains supplementary material available at 10.1007/s11306-025-02250-2.

## Introduction

In avian hatchlings the transition from stored to oral nutrition is critical to survival. The process involves adapting from stores provided by the hen to the more complex resources obtained from the environment. In chickens, the hen provides the embryo and post-hatch chicken with nutrients including lipids, proteins, vitamins, minerals and water. These resources are mostly stored in the yolk sac that is engulfed by the developing chick just prior to hatch (Freeman & Vince, 1974). The embryo also stores lipid in subcutaneous adipose tissue (Givisiez et al., 2020). Stored nutrients allow the chick to survive post-hatch for up to three days without food or water (Panda et al., 2013).

As the metabolic hub of the body (Cunningham & Porat-Shliom, 2021), the liver must adapt to changes in available nutrients. During the first six days post-hatch, the broiler (raised for meat) chicken liver exhibits positive allometric growth (Schmidt et al., 2009), indicating the liver is growing faster than the rest of the body (Gould, 1975; Huxley, 1924). By approximately day 8 post-hatch, the liver of a broiler chicken exhibits negative allometric growth (Schmidt et al., 2009). The liver is still growing, but it is now growing slower than the rest of the body. By this point, stored nutrients are largely depleted, and the liver begins its role as a metabolic hub for other organs.

The early positive allometric growth is supported by lipids stored in the yolk (Ali et al., 2007; Chamblee et al., 1992). Over the course of the first week post-hatch these lipids are depleted. In the broiler industry, feed is optimized for distinct chicken growth periods with up to four different feed formulations provided over the broiler grow out period (Pesti & Choct, 2023). These formulations all contain high protein and carbohydrate levels.

A reasonable hypothesis is that metabolites and metabolic pathways shift as nutrient availability changes post-hatch. To evaluate this hypothesis, metabolomic analysis was applied to liver samples taken from chicks every other day between days 4–20 post-hatch.(Santos et al., 2024; Wishart, 2019). Morphometric, correlation, principal component, orthogonal partial least squares discriminant analysis, cross-validation ANOVA, and network analytical methods were applied to evaluate liver growth and metabolic dynamics during this critical period.

## Methods

### Bird husbandry, necropsy and tissue collection

As described previously (Van Every & Schmidt, 2021). Day-old male Ross 708 chicks were obtained from a commercial hatchery. Standard management and husbandry procedures were followed, as approved by the Animal Care and Use Committee of the University of Delaware (IACUC 72R-2017-0). All experiments were performed in accordance with relevant guidelines and regulations. Birds were fed a commercial starter diet throughout the experiment (Southern States Cooperative, Milford DE) and had constant access to water. On each even-numbered day post-hatch from Day 4 (D4) through Day 20 (D20), 12 birds were randomly chosen and humanely euthanized by cervical dislocation. Prior to euthanasia, birds were weighed, and blood was drawn from the brachial wing vein for immediate i-STAT blood chemistry analysis using CG8 + cartridges. 200 µL. of blood was also collected in EDTA tubes on ice and then centrifuged to separate plasma for metabolome analysis. During necropsy, multiple tissues were systematically collected and snap frozen in liquid nitrogen. Select organ masses (heart, liver, breast muscle) and intestine segment lengths were also recorded. Liver was collected from the caudal portion of the left lobe, with an additional tube of tissue saved for metabolome analysis. Frozen tissues and plasma were subsequently stored at − 80 °F until further use.

### Morphometric, principal component, correlation, hierarchical clustering, and network analysis

As described previously (Van Every & Schmidt, 2021). Total mass of each bird and the mass of its corresponding liver were determined at the time of necropsy. Statistical analysis including Z-score determination, hierarchical clustering and PCA were done using JMP Pro Statistical Software (JMP^®^, version 17.1.0). Orthogonal PLS-DA, permutation analysis and CV-ANOVA were run in R-Studio using the ropls, caret, dplyr, MASS, coin and ggplot2 libraries (Kuhn, 2008; Team, 2018, 2021; Thevenot et al., 2015; Wickham, 2016; Wickham et al., 2023). Statistical cutoffs were set at an FDR < 0.05. Networks were constructed and analyzed using Cytoscape (Shannon et al., 2003).

### Metabolome

Metabolomic analysis was done as previously described (Van Every & Schmidt, 2021). Plasma and liver samples were sent to the UC Davis West Coast Metabolomics Center (Davis, CA) for untargeted metabolomic analysis. Primary metabolism analysis was done with gas chromatography-time of flight/mass spectrum (GC-TOF MS) and reported as peak heights normalized to mean total ion current, or average total metabolome levels for each tissue. A total of 657 compounds were reported. Two hundred four were identified by name and used for further analysis. Samples were extracted using the Matyash extraction procedure (Matyash et al., 2008) which includes MTBE, MeOH, and H2O. The aqueous phase was dried down and submitted to derivatization for GC. Samples are shaken at 30 C for 1.5 h then 91 µL. of MSTFA + FAMEs were added to each sample, and they are shaken at 37 C for 0.5 h to finish derivatization. Samples were then vialed, capped, and injected onto a 7890 A GC coupled with a LECO TOF. Derivatized sample (0.5 µL.) was injected using a splitless method onto a RESTEK RTX- 5SIL MS column with an Intergra-Guard at 275 C and a helium flow of 1 mL/min. The GC oven is set to hold at 50˚C for 1 min then ramped at 20˚C/min to 330˚C and then held for 5 min. The transfer line is set to 280˚C while the EI ion source is set to 250˚C. The Mass spec parameters collect data from 85 m/z to 500 m/z at an acquisition rate of 17 spectra/sec.

## Results

**Fig. 1:**
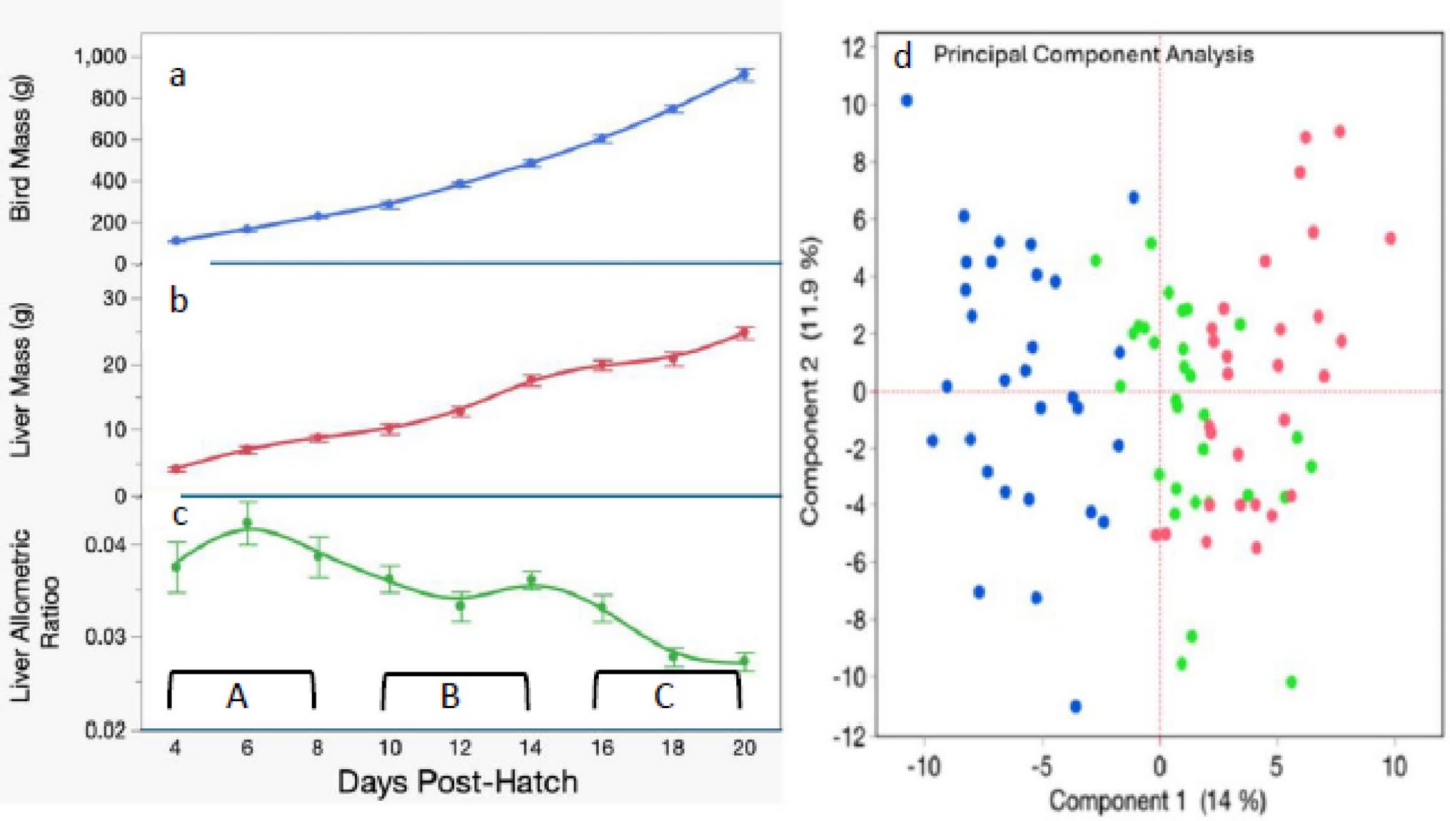
**a** Total bird mass, **b** Total liver mass, **c** Allometric liver ratio (ratio of the total liver mass to the total mass of the chicken) **A**–**C** on the X-axis refer to the periods discussed throughout the paper. **d** PCA analysis of metabolite levels in the post-hatch chicken. Blue: Days: 4,6,8. Green: Days 10,12,14; Red: Days 16,18,20. The 1 st principal component describes 14% of the variance and aligns with the three time periods suggested by the allometric liver growth curve

During the first 20 days post-hatch, bird and liver masses steadily increase, while allometric liver growth follows a complex trajectory (Fig. 1A, B, C). The allometric growth curve allows the first three weeks of post-hatch to be divided into three periods. period A spans post-hatch days 4, 6, and 8; period B spans post-hatch days 10, 12, and 14; period C spans post-hatch days 16, 18, and 20.

### Principal component analysis (PCA)

Metabolomic analysis identified and quantified 204 named metabolites. Principal component analysis (PCA) of the metabolites showed that component 1 (PC1) separates the liver by post-hatch periods (14% of the variance) (Fig. 1D). This component effectively separated the liver metabolomes of early (A) and late (C) periods, with the period B interspersed between them.

### Orthogonal-partial least squares discriminant analysis (OPLS-DA)

**Fig. 2:**
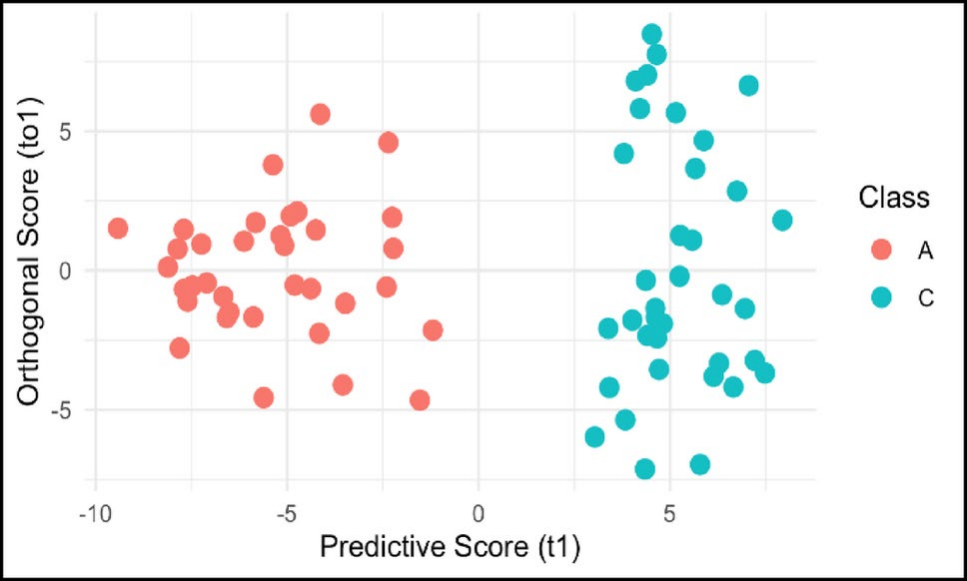
Biplot of the OPLS-DA analysis depicting separation of periods A and C by metabolites (Table 1)

The unsupervised PCA indicate that metabolites appear to distinguish between the (A) and (C) post-hatch data. To evaluate the strength of metabolites to discriminate between these periods we applied the supervised learning technique OPLS-DA (Table 1; Fig. 2). This approach achieved strong classification, with a cumulative R²Y of 0.903 indicating that 90.3% of the variance in metabolite profiles distinguishing between periods A and C is explained by the model. The model demonstrated high predictive ability with cumulative Q² values (0.859–0.861). The low Root Mean Square Error of Estimation (RMSEE = 0.159) suggests minimal error and permutation testing (1000 iterations) confirmed the significance of the model, with p-values of 0.001 for both R²Y and Q². These results indicate that OPLS-DA can effectively distinguish metabolic profiles between periods A and C.

### Cross-validation

ANOVA (CV-ANOVA) was used to evaluate further the significance of the metabolic differences between periods A and C (Table 1). This analysis yielded a highly significant F-score (651.9; *p* < 0.0001), indicating that the OPLS-DA model was unlikely influenced by overfitting. The large effect size (Eta² = 0.90) indicates that 90% of the variance in metabolite profiles can be attributed to time periods rather than random noise. The agreement between PCA, OPLS-DA, and CV-ANOVA results strongly supports a structured metabolic shift occurring across post-hatch liver development.

**Fig. 3:**
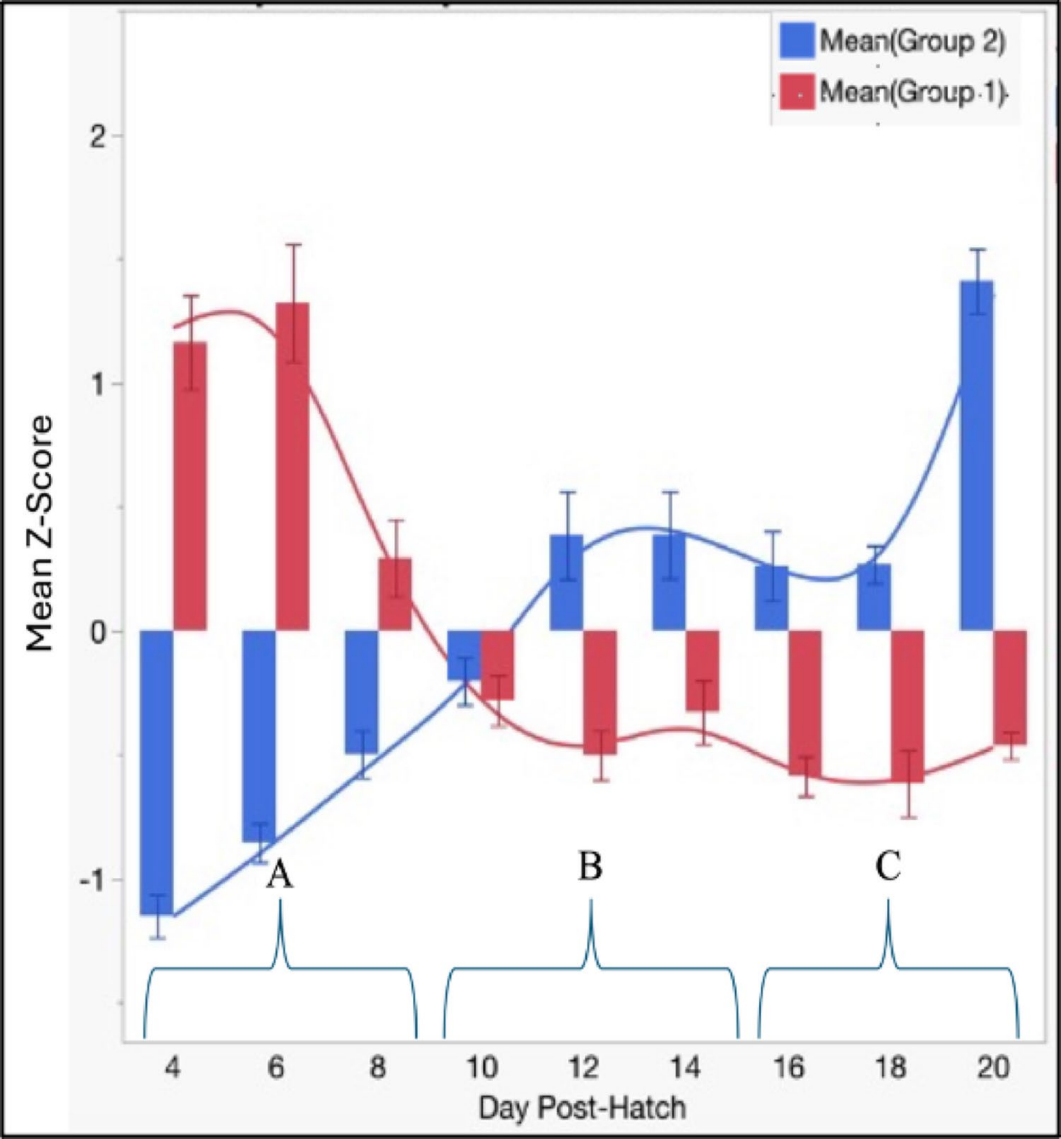
Composite Z-score plotted by day for the top five negative (Group 1 red) and five positive (Group 2, blue) contributors to the first principal component. The average Z-score was determined for the two groups of metabolites with the result plotted for *each group by day post-hatch.* Group 1 metabolites are enriched during period A, group 2 metabolites were enriched during period. Group 1 consisted of myristic acid, oleic acid, palmitic acid, 1-monopalmitin and alpha-tocopherol. Group 2 included adenine, glucose- 6-phosphate, hexose- 6-phosphate, aspartic acid and fucose

Metabolites driving the PCA and OPLS-DA analyses showed similar biochemical signatures distinguishing between periods A and C. Period A was influenced by lipid-derived metabolites, suggesting that fatty acid metabolism and antioxidant protection are important to this period (Table 2). In contrast period C exhibited a more diverse composition, characterized by a mixture of carbohydrates, amino acids, and TCA metabolites. This metabolic shift between A and C likely supports the liver as it assumes its role as the body’s metabolic center. The mean Z-score analysis of the ten metabolites contributing to PC1 in each period further supports the grouping of post-hatch liver metabolism into three periods (Fig. 3).

**Table 1:** Summary of OPLS-DA, permutation and CV-ANOVA analysis comparing metabolic profiles between early (A) and late (C) post-hatch periods

Metric	Value
R^2^Y (cumulative)	0.903
Q^2^ (cumulative)	0.858–0.861
Permutation (1000X) pR^2^Y	0.001
Permutation (1000X) pQ^2^	0.001
RMSEE	0.158
CV-ANOVA F-score (10 folds)	651.9
CV-ANOVA p-value	< 0.0001
CV-ANOVA Effect Size	Eta^2^ = 0.90

**Table 2:** Metabolites contributing to the difference between period A and C

Metabolite	Period	VIP	Log2_FC	FDR	CohenD	Random Forest		F.Ratio
fucose	C	2.197	0.315	0.0001	− 2.981	10.948	158.7	
palmitoleic acid	A	2.088	0.6207	0.0001	2.6017	10.738	118	
adenine	C	2.166	0.4506	0.0001	− 2.654	7.5464	122.4	
threonic acid	C	2	0.3464	0.0001	− 2.402	9.6271	100.4	
erythronic acid lactone	C	1.954	0.3371	0.0001	− 2.221	8.9102	87.25	
myristic acid	A	1.775	0.7718	0.0001	1.8929	4.8933	61.97	
alpha-tocopherol	A	1.596	0.8687	0.0001	1.52	9.8643	39.54	
palmitic acid	A	1.799	0.696	0.0001	1.907	4.0182	64.41	
malic acid	C	1.566	− 0.641	0.0001	− 1.449	9.2976	36.84	
fumaric acid	C	1.578	− 0.627	0.0001	− 1.443	9.0906	36.49	
aspartic acid	C	1.772	0.6309	0.0001	− 1.753	3.6513	52.96	
oleic acid	A	1.736	0.3108	0.0001	1.723	4.3014	51.24	
1-monopalmitin	A	1.683	0.6492	0.0001	1.6593	3.3109	49.58	
ribonic acid	C	1.747	0.0127	0.0001	− 1.665	5.2079	48.62	
threonine	A	1.819	− 0.079	0.0001	1.7673	2.9442	54.94	
inosine	C	1.788	0.1633	0.0001	− 1.74	2.6601	52.27	
uric acid	A	1.788	− 0.012	0.0001	1.7048	3.5922	49.99	
lysine	A	1.633	0.1214	0.0001	1.508	6.2052	40.57	
2,5-dihydroxypyrazine	C	1.709	0.2083	0.0001	− 1.602	3.5953	46.02	
uracil	A	1.717	0.1965	0.0001	1.6384	3.0358	46.14	
nicotinamide	C	1.569	0.7942	0.0001	− 1.436	3.393	35.81	
gamma-tocopherol	A	1.611	0.4837	0.0001	1.4741	2.8894	37.08	
glucose	C	1.565	− 0.324	0.0001	− 1.504	3.272	39.59	
uridine	C	1.55	0.1913	0.0001	− 1.499	3.8239	38.92	
glutaric acid	A	1.573	− 0.283	0.0001	1.4361	3.6851	35.53	
glucose- 6-phosphate	C	1.558	− 0.08	0.0001	− 1.45	4.2692	36.75	
hypoxanthine	C	1.519	0.6311	0.0001	− 1.35	2.3655	31.16	
maleimide	C	1.459	0.8709	0.0001	− 1.264	2.0582	27.5	
fructose- 6-phosphate	C	1.542	− 0.175	0.0001	− 1.405	2.7761	34.59	
N-acetylornithine	A	1.538	0.1857	0.0001	1.3797	2.8875	34.69	
oxoproline	C	1.387	0.8074	0.0001	− 1.187	2.8285	25.18	
hexose- 6-phosphate	C	1.5	0.0666	0.0001	− 1.337	3.4767	31.26	
2,4-diaminobutyric acid	C	1.254	1.2477	0.0001	− 1.078	2.2624	20.25	
tyrosine	A	1.498	0.0523	0.0001	1.3097	2.4754	30.91	
9-myristoleate	C	1.091	2.5447	0.0011	− 0.889	1.2955	13.15	
dodecanol	A	1.294	0.9627	0.0001	1.1352	1.5767	21.99	
kynurenine	A	1.337	1.0545	0.0001	1.1381	0.4751	22.47	
guanosine	C	1.402	0.6786	0.0001	− 1.164	0.5745	23.33	
linoleic acid	A	1.369	0.788	0.0001	1.1971	− 0.108	24.79	
phenylethylamine	C	1.316	− 0.44	0.0001	− 1.082	2.6634	19.86	
hydroxylamine	A	1.227	0.7388	0.0001	1.049	2.3766	18.87	
methanolphosphate	C	1.344	− 0.192	0.0001	− 1.21	2.3052	25.65	
hexitol	C	1.333	− 0.336	0.0001	− 1.2	1.5854	27.27	
maltose	A	1.393	0.8158	0.0001	1.169	− 1.042	23.62	
asparagine	C	1.089	2.5202	0.0016	− 0.86	− 0.55	13.55	
sorbitol	C	1.384	− 0.042	0.0001	− 1.279	0.2559	27.86	
cis-gondoic acid	A	1.249	− 0.409	0.0001	1.0533	1.8153	18.64	
stearic acid	A	1.189	0.3792	0.0002	1.0451	2.2192	18.61	
fructose	C	1.299	0.0674	0.0001	− 1.183	0.9819	24.47	
5-hydroxy- 3-indoleacetic acid	C	1.199	0.4643	0.0003	− 0.993	1.9237	16.62	
cystine	C	1.297	− 0.276	0.0001	− 1.137	0.0016	22.18	
xylitol	C	1.157	0.9321	0.0004	− 0.973	0.4473	15.79	
2-deoxytetronic acid	C	1.21	0.6227	0.0003	− 0.994	0.1869	16.74	
fructose- 1-phosphate	C	1.179	− 0.492	0.0005	− 0.976	1.7464	15.92	
parabanic acid	C	1.153	− 0.422	0.0006	− 0.966	1.8386	16.85	
UDP-glucuronic acid	C	1.159	0.3273	0.0004	− 0.975	1.2575	16.5	
maltotriose	A	1.147	0.6551	0.0009	0.914	1.3643	14.84	
beta-glycerolphosphate	C	1.111	1.1385	0.0011	− 0.887	0	13.73	
myo-inositol	A	1.116	0.4268	0.0008	0.9178	1.9778	14.26	
alpha-ketoglutarate	C	1.046	− 1.219	0.0016	− 0.861	1.5797	13.75	
3-phenyllactic acid	A	1.197	0.2142	0.0009	0.9164	1.6613	13.73	
N-acetyl- 5-hydroxytryptamine	A	1.078	0.5157	0.0009	0.9022	1.138	15.42	
alanine	A	1.10	0.4176	0.001	0.8982	1.412	13.84	
putrescine	A	1.159	0.0905	0.0007	0.9528	0.2583	16.34	
3-phosphoglycerate	A	1.212	− 0.231	0.0009	0.91	0.0258	13.54	
glutamic acid	C	1.135	0.3536	0.0017	− 0.857	2.8289	13.32	
isocitric acid	C	1.114	0.6916	0.0018	− 0.875	0.9	13.22	
glycerol- 3-galactoside	A	1.076	1.2421	0.0021	0.8346	− 0.822	12.95	
glycyl proline	A	1.066	0.9135	0.0025	0.8443	1.3733	13.67	
gluconic acid lactone	C	1.014	− 0.388	0.0017	− 0.854	− 0.108	12.65	
proline	A	1.054	0.4862	0.0026	0.8268	1.6086	13.01	
dehydroascorbic acid	C	1.049	− 0.471	0.0023	− 0.828	− 0.336	12.97	
glutamine	C	1.099	0.8158	0.0035	− 0.793	1.1228	10.97	
conduritol-beta-epoxide	A	1.023	− 1.031	0.0042	0.7798	− 0.61	9.755	
phosphoenolpyruvate	A	1.047	0.0608	0.004	0.7879	− 0.57	10.05	

The period column indicates the period in which the metabolite is enriched. Metabolites were selected based on the variable importance of projection (VIP > 1) as determined from the OPLSDA test. Log2 FC is the log base 2 of the fold difference as determined by A/C. FDR is the false discovery rate, CohenD was determined from the CV-ANOVA, Random Forest values indicate the Gini Importance value, F statistic was determined by the CV-ANOVA.

### Network analysis

**Fig. 4:**
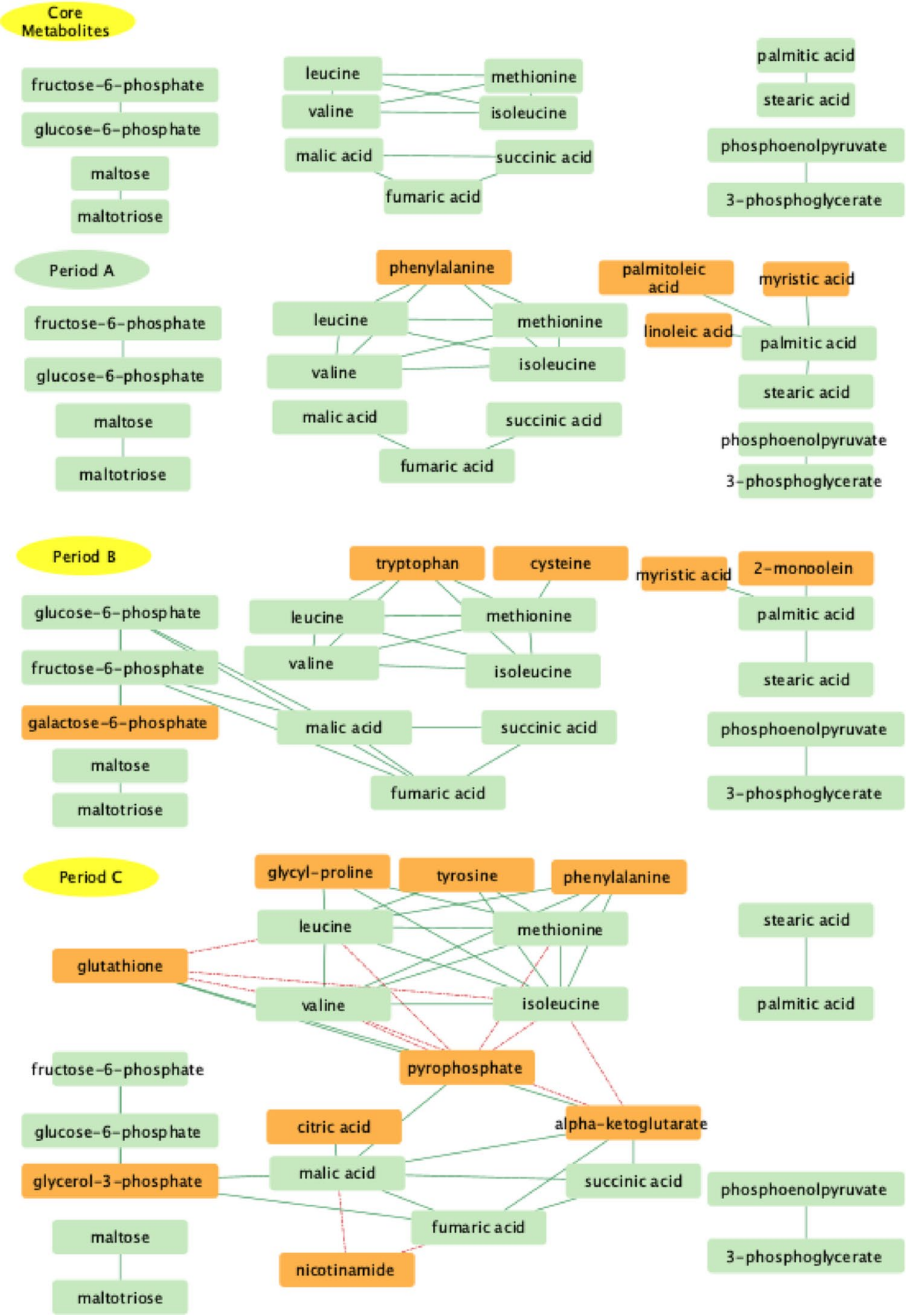
Networks of metabolites representing the core metabolites whose correlations are maintained across all three growth periods (Corr > 0.85; FDR < 0.05) Period A, Period B, and Period C depict the core metabolites and the first neighbors that appear in each period

To identify changes in metabolite relationships over time, networks were constructed in Cytoscape (Shannon et al., 2003) based on metabolite correlation coefficients (Table S1). Fifteen metabolites retained strong correlations across all three time periods, (FDR < 0.05) (Fig. 4 Core Metabolites) Core amino acid metabolites include the three branched chain amino acids, valine, isoleucine and leucine, along with methionine. Glycolysis is represented by two metabolites from phase 1 (ATP investment phase), glucose- 6- phosphate and fructose- 6-phosphate along with two from phase 2 (ATP return phase), 3-phosphoglycerate and phosphoenolpyruvate. Starch derived maltose and maltotriose maintained correlation across all three periods. These are processed through the glycolytic pathway following conversion of maltotriose to maltose and maltose to two glucose molecules. The TCA cycle metabolites retaining correlation across these periods are succinic acid, fumaric acid and malic acid. Lipid metabolism is represented by palmitic and stearic acid. Palmitic acid is central to both lipid beta-oxidation and lipid elongation while stearic acid is the first elongation product of palmitic acid.

### First neighbors

Network analysis was extended by identifying first neighbors of the core metabolites (Fig. 3: Periods A-C). This analysis focused on major metabolic pathways including the TCA cycle, amino and fatty acid metabolism, and glycolysis and gluconeogenesis. The metabolites during period A form pathway specific clusters, suggesting that the pathways are functioning independently. In period B, phase 1 glycolytic metabolites and the TCA cycle have connected with edges between glucose- 6-phosphate and fructose- 6-phosphate to fumaric acid and malic acid. In period C, phase 1 of glycolysis remains linked to the TCA cycle via glycerol- 3-phosphate. The TCA cycle expands to include citric acid and alpha ketoglutarate (αKG). αKG connects the TCA cycle to the amino acid cluster, exhibiting negative correlations with isoleucine and valine. In addition, pyrophosphate and glutathione provide further interactions, exhibiting positive correlations with metabolites of the TCA cycle but negative correlations with amino acid metabolism.

**Table 3:** Network characteristics for core metabolites of periods A, B, C as determined by cytoscape

Network Parameter	Period A	Period B	Period C
Number of nodes:	19	20	25
Number of edges:	19	26	47
Avg. number of neighbors:	4	3.333	4.632
Network diameter:	1	3	6
Network radius:	1	2	3
Characteristic path length:	1	1.4	2.52
Clustering coefficient:	1	0.778	0.661
Network density:	1	0.667	0.257
Network heterogeneity:	0	0.283	0.476
Network centralization:	0	0.2	0.271
Connected components:	6	5	4

### Network parameters

These qualitative observations from the graphs were supported by examining the quantitative network parameters (Table 3). The edge, node and average number of neighbor parameters all increase as the liver proceeds from period A to period C. This indicates that the overall complexity of the metabolism is increasing with time. Path length, network diameter and network radius also increase with liver maturation. Collectively these metrics indicate metabolism is developing greater metabolic capacity as more links have arisen between pathways. In addition, both network heterogeneity and centralization increase, consistent with an increase in hub metabolites as the system matures. In contrast, clustering coefficient, network density and connected components all decrease during this time. This last group further supports the emergence of hubs and a concomitant decrease in number of independent modules.

### Liver allometric ratio and total liver growth

**Fig. 5:**
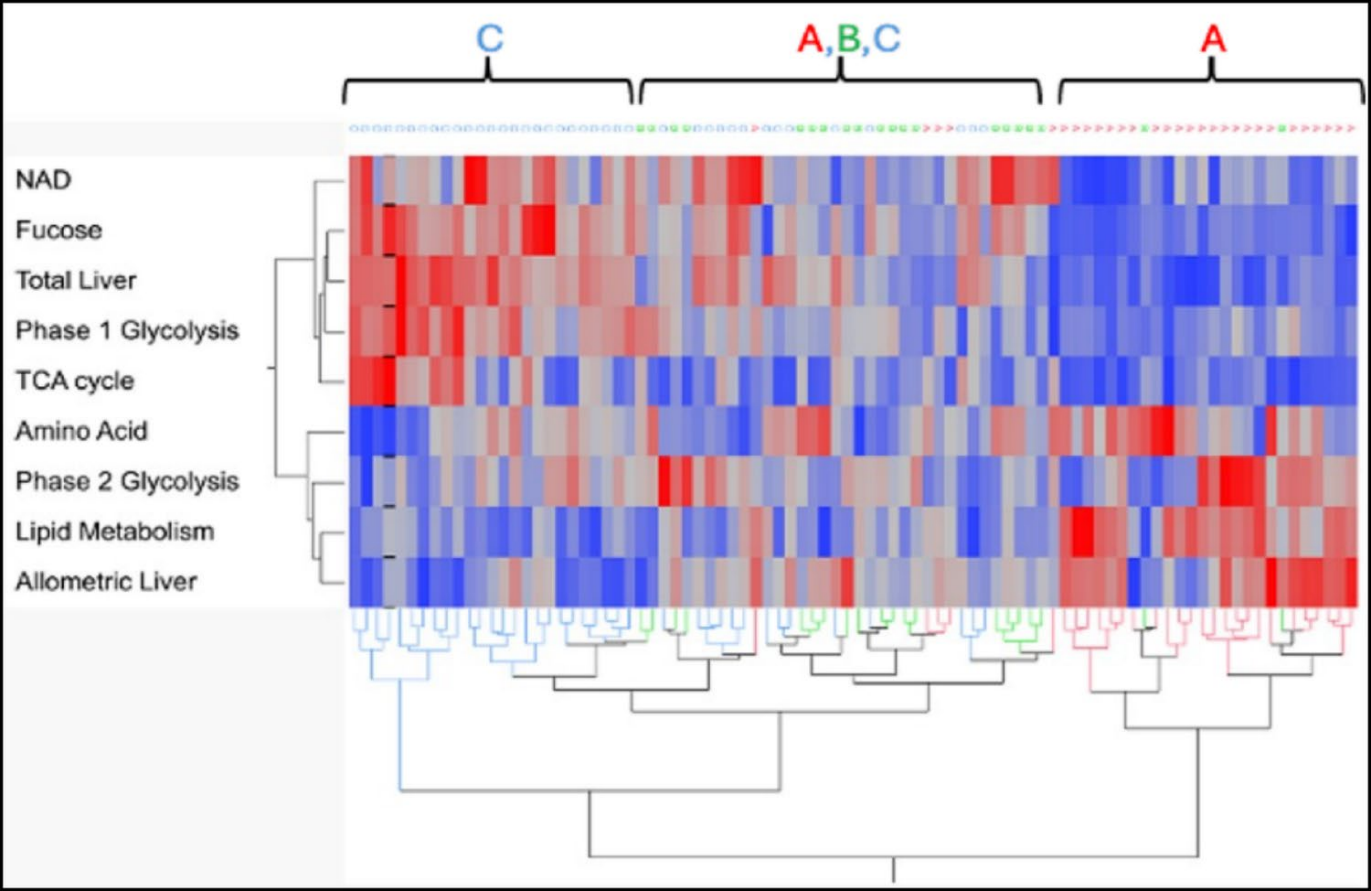
A heat map representing results of hierarchical clustering of metabolic pathways and their relation to total and allometric liver growth. NAD levels are indicative of oxidative and reductive levels in the tissue, while fucose is indicative of glycoprotein production. A, B and C refer to the time periods days 4–8, 10–14, and 16–20 respectively. Red indicates greater levels of metabolites related to the identified pathways, while blue indicates lower levels. A and C identify regions of the heat map where samples from these respective periods cluster. The middle portion of the plot is a mixture of samples from periods A, B and C

The liver’s allometric growth decreases as total liver growth increases, suggesting the two may respond to distinct metabolic signals. Metabolites associated with allometric liver growth may help explain the dynamics of allometric scaling. Pairwise correlation analysis identified metabolites exhibiting positive correlation with allometric liver growth (FDR < 0.05; 4). The majority of these are lipids along with the vitamin E compounds alpha- and gamma-tocopherol. Also associated with allometric growth are the phase 2 glycolytic metabolites 3-phosphoglycerate and phosphoenolpyruvate, along with glycerol and the amino acids lysine and threonine. Metabolites of glycolysis phase 1 and TCA cycle exhibit negative correlation with allometric growth. Metabolites exhibiting positive correlation with total liver growth include components of glycolysis phase 1, lipid metabolism, the TCA cycle, fructose metabolism, and amino acids. Nicotinamide, a precursor to NAD + is also positively correlated with total liver growth, along with fucose, a component of sugar residues of many glycoproteins and glycolipids. Other metabolites exhibiting positive association with total liver growth include glycerol- 3-phosphate, the amino acid aspartic acid and the lipid lauric acid. Figure 5 summarizes the temporal relationships between pathways and allometric or total liver growth.

### Xenometabolites

Metabolomics identified several metabolites found in the liver that are likely xenometabolites. 1,2,4-benzenetriol, benzoic acid, and pyrogallol are probably derived from corn used for feed. Methanolphosphate may have originated from feed grain fungal contaminants. Nornicotine is a metabolite of nicotine. To our knowledge tobacco is not used in the poultry industry suggesting this is an environmental contaminant. 2-piperidinobenzonitrile is used to synthesize pesticides and herbicides. Levoglucosan is produced by pyrolysis of cellulose and is found in biochar, a substance occasionally used in chicken feed. Maleimide and terephthalic acid are commonly used in plastic synthesis and may appear due to pervasive microplastic contaminants. The sources of 2,5-dihydroxypyrazine, 1,3,5-trimethylcyanuric and conduritol-beta-epoxide are uncertain.

## Discussion

Metabolic network changes across periods A, B and C provide insight into post-hatch liver maturation. Lipids provide a strong contribution to period A (days 4–8) supporting positive allometric growth under normoxic and hypoxic conditions (Corpechot et al., 2002; Nath & Szabo, 2012; Van Every & Schmidt, 2021). During period B (days 10–14) maternal resources are being depleted (Cook & Bletner, 1955) as metabolism shifts to relying on nutrients from feed (Fig. 3). Period C exhibits properties of the mature liver with metabolism geared to provide nutrients for other tissues. In addition, fucose makes a major contribution to period C by supporting the liver’s role in synthesizing serum glycoproteins such as complement proteins.

Network analysis identified fifteen metabolites common to periods A, B, and C, forming clusters related to glycolysis and gluconeogenesis, the TCA cycle, along with lipid and amino acid metabolism (Figs. 4 and 5). This preservation implies that regulatory mechanisms during the first 20 days post-hatch actively maintain these metabolic relationships. The clusters define critical metabolites within pathways that are essential to the maturing liver’s homeostatic core functions. These core functions shift as the liver matures, necessitating increased network connectivity during periods B and C. Evidence of liver functional maturation is supported by glucose- 6-phosphatase (G6P) transcript enrichment at day 20 compared to day 4 (Van Every & Schmidt, 2021). G6P activity is essential to the mature liver as it allows glucose export for use by other tissues.

This functional maturation is also evidenced in the quantitative network parameters (Table 3). Period A consists of tight modules with the pathways of core metabolism operating independently. As the liver transitions to period B, there is an increase in the number of nodes and new edges appear connecting different pathways. Although the number of connections grows in Period B, the network expands faster than it connects, resulting in lower density and clustering. This suggests the period B network expands without a proportional increase in the number of edges. Finally, period C core network parameters are consistent with a mature, resilient metabolism. This is emphasized by an almost doubling of the number of edges. These additional edges reflect the strong correlations between metabolites of different pathways. In combination, the network characterization indicate that period A contains an independently operating core metabolism, period B is a transitional phase with period C exhibiting an integrated mature core metabolic network.

Glycolysis phase 1 and 2 remain independent across the post-hatch periods. Glycolysis phase 1 can supply metabolites to phase 2 for ATP production. Alternatively, it can shunt metabolites to the pentose phosphate pathway (NADPH and nucleotide synthesis), the glucosamine pathway (glycoproteins), or to serine production. Phase 2 can operate independently by importing glycerol, fructose or pentose phosphate metabolites to drive ATP production. Phase 2 metabolites can also be shunted to lactate for NAD + regeneration, used to synthesize alanine and the branched chain amino acids, or to acetyl-CoA for lipid synthesis. The continued separation of these phases provides the liver with metabolic flexibility by balancing the need for glycolytic ATP production with the need to synthesize essential biomolecules.

The TCA cycle cluster consists of malic acid, succinic acid, and fumaric acid with citric acid and α-ketoglutarate joining during period C. Citric acid is the first product of the TCA cycle and α-ketoglutarate is important to the cycle because it can be synthesized from glutamate. The expanded network in period C could result from the mature liver’s need for stable TCA cycle flux to support its own nutrient needs and those of other tissues.

The core fatty acid network cluster is composed of palmitic and stearic acid. These are versatile lipids that can either provide energy or be used to synthesize other lipids and triacylglycerols. Shifts in their first neighbors are indicative of the liver’s metabolic maturation. During period A the cluster also contains palmitoleic acid, myristic acid, and linoleic acid supporting positive allometric growth by providing energy and membrane lipids. During period B, 2-monoolein and myristic acid are added emphasizing the increasing need to synthesize triacylglycerols. In period C, only the core metabolites palmitic and stearic acid are present. The reduction to these core lipid components may result from the need for metabolic flexibility as the liver supports its own and systemic nutrient demands.

The amino acid core metabolites consist of the branched-chain amino acids leucine, isoleucine and valine along with methionine. The branched chain amino acids are likely preserved for the energy needs of other tissues due to low levels of liver Branched-Chain Aminotransferase. Methionine is required for incorporation into proteins, and for the synthesis of S-adenosylmethionine and cysteine. A significant addition to the core amino acids in period B is tryptophan. Tryptophan catabolism produces metabolites that regulate immune system function and tolerance (Seo & Kwon, 2023; Stone & Williams, 2023). As period B coincides with expansion of the liver’s immune system (Van Every and Schmidt, unpublished) tryptophan may be crucial to establishing tolerance to feed antigens and the microbiome. Period C adds phenylalanine, tyrosine, and glycyl-proline to the core amino acids. For catabolism, phenylalanine is converted to tyrosine which is then metabolized to acetoacetic acid and fumaric acid for energy. Glycyl-proline is a dipeptide derived from collagen proteolysis. Collagen turnover is important to regulating interactions between hepatocytes and non-parenchymal cells during this period.

**Fig. 6:**
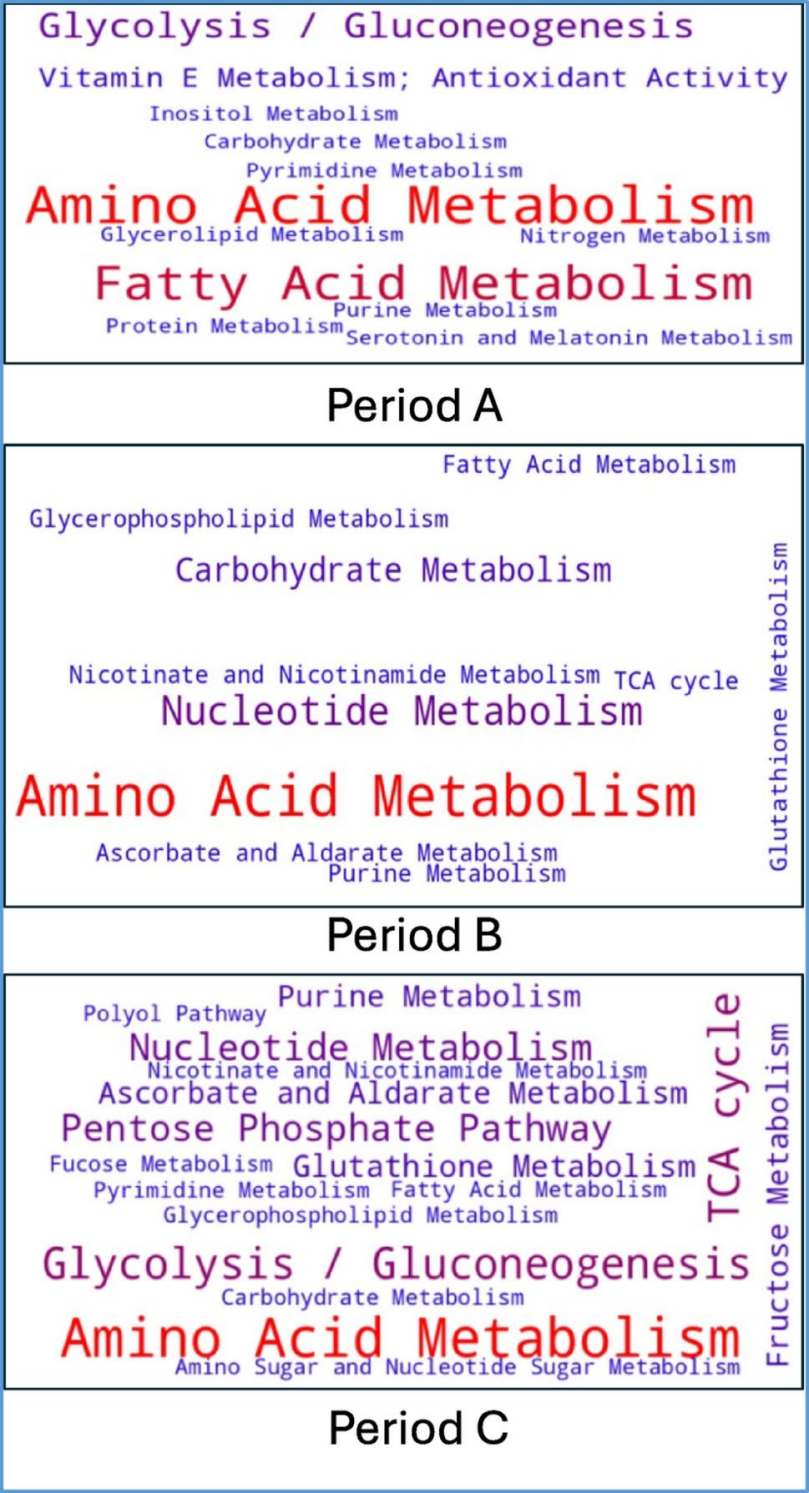
Word clouds summarizing the dominant metabolic pathways during liver development across post-hatch periods **A** (days 4–8), **B** (days 10–14), and **C** (days 16–20). Each word cloud represents pathways enriched during the respective time periods based on metabolomic analysis and pathway annotation of metabolites. Font size reflects the relative prominence or enrichment of each pathway

The word clouds (Fig. 6) provide a visual summary of the metabolic pathways that dominate during periods A, B, and C. Period A is dominated by of amino acid and fatty acid metabolism consistent with the liver’s reliance on maternal lipids and amino acids to support rapid post-hatch growth. Glycolysis/gluconeogenesis is also present emphasizing the liver’s early role in energy production. In Period B, amino acid metabolism continues its importance along with increasing emphasis on nucleotide metabolism, carbohydrate metabolism, and TCA cycle activity. This period is less populated by pathways, perhaps due to its role as a transition from periods A to C. The period C word cloud contains more pathways than either A or B. Glycolysis/gluconeogenesis, amino acid metabolism and TCA cycle predominate but they have been joined by several other pathways such as the pentose phosphate pathway and glutathione metabolism. This expansion during period C is integral to the liver’s maturation into an organ capable of supporting systemic and its own metabolic needs. This shift toward metabolic diversity and resilience is consistent with the network analysis showing increased connectivity and complexity.

Morphometric analyses of the days 4–20 shows a constant increase in total liver mass, but a complex allometric growth curve. Early post hatch, the liver exhibits positive allometric growth to provide sufficient capacity to support the increasing nutrient demands. Subsequently the liver shifts to negative allometric growth between days 6–10. There is a brief return to positive allometric growth between days 12–14, which then returns to negative for the remainder of the days (Schmidt et al., 2009). Allometric growth is driven by lipid metabolism, glycolysis phase 2, and the amino acids threonine and lysine. In oxygen rich regions, lipids can provide the energy needed to drive proliferation and to enlarge the membrane. In hypoxic regions, the second phase of glycolysis provides the necessary energy with lipids supporting membrane integrity. Threonine and lysine stimulate the Mechanistic Target of Rapamycin complex 1 (MTOR1), a potent activator of cellular proliferation (Jang et al., 2020; Ryu & Han, 2011). By regulating allometric growth, metabolism maintains appropriate proportions between liver and body mass.

Pathways and specific metabolites associated with total liver growth also support systemic metabolic needs. These include glycerol- 3-phosphate, lauric acid, the TCA cycle, phase 1 of glycolysis, and the amino acids glutamic acid and glutamine. Glycerol- 3-phosphate is essential to triacylglycerol synthesis and it can be used for gluconeogenesis. Lauric acid is a 12-carbon saturated fat that can bypass the mitochondrial carnitine shuttle. Consequently, lauric acid typically undergoes mitochondrial oxidation to support the TCA cycle and ATP production. The TCA cycle provides reducing equivalents for the electron transport chain or biosynthetic precursors to other pathways. Glutamic acid can sustain the TCA cycle by conversion to alpha-ketoglutarate or used in the synthesis of glutathione and nucleotides. Glutamine is essential to nitrogen balance and detoxification of ammonia.

In addition to its role in metabolic homeostasis, the liver is the primary site for detoxification. This is relevant given the detection of xenometabolites in post-hatch liver samples, indicating potential environmental and dietary exposures that may influence metabolic processes. For example, 1,2,4-benzenetriol and pyrogallol are likely derived from feed and are associated with oxidative stress and DNA damage. Methanolphosphate suggests feed fungal contamination possibly indicating the presence of other toxic fungal metabolites. Nornicotine is likely due to environmental contamination, perhaps from smokers. 2-piperidinobenzonitrile, a compound used in pesticide and herbicide synthesis is likely due to the use of these agents on our farm. Additionally, maleimide and terephthalic acid, which are associated with plastic production, raise concerns about microplastic exposure. Identifying these xenometabolites suggests a need for examining the bioavailability, accumulation, and physiological effects of these compounds in poultry, as well as potential implications for food safety and human health.

Central mechanisms in the brain respond to environmental and internal stimuli with changes in secreted hormones and growth factors to control systemic cell growth. The liver complements this information by monitoring metabolites and xenometabolites from the intestine (via the portal vein) and systemic metabolic levels (via the hepatic artery). Integrating neuronal and metabolic signals can control the liver’s proportional size as the body grows post-hatch. As the liver matures, increased network connectivity allows greater exchange of metabolites between pathways. This connectivity buffers metabolic fluctuations, enhancing resilience and stability between fed and fasting states. For future efforts, these insights provide hypotheses regarding gene expression dynamics that drive or respond to shifts in metabolic resources. Overall, this study provides a foundation for how metabolic networks impact liver growth from the post-hatch period to the juvenile stage.

## Supplementary Information

Below is the link to the electronic supplementary material.

Supplementary material 1 (DOCX 23.1 kb)

## Data Availability

The metabolomics and metadata reported in this paper are available via the Metabolomics Workbench (Sud et al., 2016) study identifier: project: PR002272, datatrack_id:5413, study_id: ST003663. The DOI for this is: http://dx.doi.org/10.21228/M8Z54 T.
