# Supplementary material for: Metabolic and morphometric analysis of allometric and total liver growth in Post-Hatch chickens
Source: Metabolomics. 2025 Apr 12;21(3):52. doi: 10.1007/s11306-025-02250-2 (PMC11993442; doi:10.1007/s11306-025-02250-2)
Supplement: Supplementary file 1 — Supplementary material 1 (DOCX 23.1 kb) [file 11306_2025_2250_MOESM1_ESM.docx]

| Supplemental Table. Cytoscape edge table showing metabolite pairs used to generate Fig. 4. IF Negative 1 = negative correlation; 0 = positive correlation. | | | | | |
| --- | --- | --- | --- | --- | --- |
| Correlation | FDR Adj P-Value | IF Negative | interaction | Metabolite Pairs | Period |
| 0.9504 | < 0.0001 | 0 | interacts with | 3-phosphoglycerate (interacts with) phosphoenolpyruvate | C |
| 0.8265 | < 0.0001 | 0 | interacts with | fructose-6-phosphate (interacts with) glucose-6-phosphate | C |
| 0.9549 | < 0.0001 | 0 | interacts with | fumaric acid (interacts with) malic acid | C |
| 0.7988 | < 0.0001 | 0 | interacts with | fumaric acid (interacts with) succinic acid | C |
| 0.9401 | < 0.0001 | 0 | interacts with | isoleucine (interacts with) leucine | C |
| 0.8844 | < 0.0001 | 0 | interacts with | isoleucine (interacts with) methionine | C |
| 0.938 | < 0.0001 | 0 | interacts with | isoleucine (interacts with) valine | C |
| 0.939 | < 0.0001 | 0 | interacts with | leucine (interacts with) methionine | C |
| 0.9695 | < 0.0001 | 0 | interacts with | leucine (interacts with) valine | C |
| 0.803 | < 0.0001 | 0 | interacts with | malic acid (interacts with) succinic acid | C |
| 0.8933 | < 0.0001 | 0 | interacts with | maltose (interacts with) maltotriose | C |
| 0.9206 | < 0.0001 | 0 | interacts with | methionine (interacts with) valine | C |
| 0.7724 | < 0.0001 | 0 | interacts with | palmitic acid (interacts with) stearic acid | C |
| 0.9524 | < 0.0001 | 0 | interacts with | isoleucine (interacts with) leucine | A |
| 0.8912 | < 0.0001 | 0 | interacts with | isoleucine (interacts with) methionine | A |
| 0.8416 | < 0.0001 | 0 | interacts with | isoleucine (interacts with) phenylalanine | A |
| 0.9091 | < 0.0001 | 0 | interacts with | isoleucine (interacts with) valine | A |
| 0.9593 | < 0.0001 | 0 | interacts with | leucine (interacts with) valine | A |
| 0.9376 | < 0.0001 | 0 | interacts with | leucine (interacts with) methionine | A |
| 0.8041 | < 0.0001 | 0 | interacts with | leucine (interacts with) phenylalanine | A |
| 0.8121 | < 0.0001 | 0 | interacts with | maltose (interacts with) maltotriose | A |
| 0.8132 | < 0.0001 | 0 | interacts with | methionine (interacts with) phenylalanine | A |
| 0.8723 | < 0.0001 | 0 | interacts with | methionine (interacts with) valine | A |
| 0.7727 | < 0.0001 | 0 | interacts with | linoleic acid (interacts with) palmitic acid | A |
| 0.7776 | < 0.0001 | 0 | interacts with | palmitic acid (interacts with) palmitoleic acid | A |
| 0.7069 | < 0.0001 | 0 | interacts with | palmitic acid (interacts with) stearic acid | A |
| 0.9351 | < 0.0001 | 0 | interacts with | myristic acid (interacts with) palmitic acid | A |
| 0.7347 | < 0.0001 | 0 | interacts with | phenylalanine (interacts with) valine | A |
| 0.9251 | < 0.0001 | 0 | interacts with | 3-phosphoglycerate (interacts with) phosphoenolpyruvate | A |
| 0.7443 | < 0.0001 | 0 | interacts with | fumaric acid (interacts with) malic acid | A |
| 0.7538 | < 0.0001 | 0 | interacts with | fumaric acid (interacts with) succinic acid | A |
| 0.8069 | < 0.0001 | 0 | interacts with | fructose-6-phosphate (interacts with) glucose-6-phosphate | A |
| 0.7806 | < 0.0001 | 0 | interacts with | 3-phosphoglycerate (interacts with) phosphoenolpyruvate | B |
| 0.7506 | < 0.0001 | 0 | interacts with | fructose-6-phosphate (interacts with) fumaric acid | B |
| 0.8199 | < 0.0001 | 0 | interacts with | fructose-6-phosphate (interacts with) galactose-6-phosphate | B |
| 0.9195 | < 0.0001 | 0 | interacts with | fructose-6-phosphate (interacts with) glucose-6-phosphate | B |
| 0.7163 | < 0.0001 | 0 | interacts with | fructose-6-phosphate (interacts with) malic acid | B |
| 0.7658 | < 0.0001 | 0 | interacts with | fumaric acid (interacts with) glucose-6-phosphate | B |
| 0.9801 | < 0.0001 | 0 | interacts with | fumaric acid (interacts with) malic acid | B |
| 0.7354 | < 0.0001 | 0 | interacts with | fumaric acid (interacts with) succinic acid | B |
| 0.7977 | < 0.0001 | 0 | interacts with | galactose-6-phosphate (interacts with) glucose-6-phosphate | B |
| 0.7197 | < 0.0001 | 0 | interacts with | tryptophan (interacts with) valine | B |
| 0.7397 | < 0.0001 | 0 | interacts with | glucose-6-phosphate (interacts with) malic acid | B |
| 0.9678 | < 0.0001 | 0 | interacts with | isoleucine (interacts with) leucine | B |
| 0.9437 | < 0.0001 | 0 | interacts with | isoleucine (interacts with) methionine | B |
| 0.776 | < 0.0001 | 0 | interacts with | isoleucine (interacts with) tryptophan | B |
| 0.9561 | < 0.0001 | 0 | interacts with | isoleucine (interacts with) valine | B |
| 0.9698 | < 0.0001 | 0 | interacts with | leucine (interacts with) methionine | B |
| 0.7778 | < 0.0001 | 0 | interacts with | leucine (interacts with) tryptophan | B |
| 0.9789 | < 0.0001 | 0 | interacts with | leucine (interacts with) valine | B |
| 0.7144 | < 0.0001 | 0 | interacts with | malic acid (interacts with) succinic acid | B |
| 0.8715 | < 0.0001 | 0 | interacts with | maltose (interacts with) maltotriose | B |
| 0.7902 | < 0.0001 | 0 | interacts with | methionine (interacts with) tryptophan | B |
| 0.9457 | < 0.0001 | 0 | interacts with | methionine (interacts with) valine | B |
| 0.7356 | < 0.0001 | 0 | interacts with | cysteine (interacts with) methionine | B |
| 0.7154 | < 0.0001 | 0 | interacts with | 2-monoolein (interacts with) palmitic acid | B |
| 0.7825 | < 0.0001 | 0 | interacts with | palmitic acid (interacts with) stearic acid | B |
| 0.8146 | < 0.0001 | 0 | interacts with | myristic acid (interacts with) palmitic acid | B |
| 0.9206 | < 0.0001 | 0 | interacts with | methionine (interacts with) valine | C |
| 0.701 | < 0.0001 | 0 | interacts with | methionine (interacts with) tyrosine | C |
| -0.7524 | < 0.0001 | 1 | interacts with | methionine (interacts with) pyrophosphate | C |
| 0.7772 | < 0.0001 | 0 | interacts with | methionine (interacts with) phenylalanine | C |
| 0.8933 | < 0.0001 | 0 | interacts with | maltose (interacts with) maltotriose | C |
| 0.7054 | < 0.0001 | 0 | interacts with | citric acid (interacts with) malic acid | C |
| 0.803 | < 0.0001 | 0 | interacts with | malic acid (interacts with) succinic acid | C |
| 0.7295 | < 0.0001 | 0 | interacts with | malic acid (interacts with) pyrophosphate | C |
| -0.7447 | < 0.0001 | 1 | interacts with | malic acid (interacts with) nicotinamide | C |
| 0.746 | < 0.0001 | 0 | interacts with | leucine (interacts with) tyrosine | C |
| -0.8066 | < 0.0001 | 1 | interacts with | leucine (interacts with) pyrophosphate | C |
| 0.7822 | < 0.0001 | 0 | interacts with | leucine (interacts with) phenylalanine | C |
| 0.939 | < 0.0001 | 0 | interacts with | leucine (interacts with) methionine | C |
| 0.9695 | < 0.0001 | 0 | interacts with | leucine (interacts with) valine | C |
| 0.938 | < 0.0001 | 0 | interacts with | isoleucine (interacts with) valine | C |
| 0.7818 | < 0.0001 | 0 | interacts with | isoleucine (interacts with) tyrosine | C |
| -0.7657 | < 0.0001 | 1 | interacts with | isoleucine (interacts with) pyrophosphate | C |
| 0.785 | < 0.0001 | 0 | interacts with | isoleucine (interacts with) phenylalanine | C |
| 0.8844 | < 0.0001 | 0 | interacts with | isoleucine (interacts with) methionine | C |
| 0.9401 | < 0.0001 | 0 | interacts with | isoleucine (interacts with) leucine | C |
| 0.7528 | < 0.0001 | 0 | interacts with | glycyl proline (interacts with) methionine | C |
| 0.7102 | < 0.0001 | 0 | interacts with | glycyl proline (interacts with) leucine | C |
| 0.704 | < 0.0001 | 0 | interacts with | glycyl proline (interacts with) isoleucine | C |
| 0.7601 | < 0.0001 | 0 | interacts with | glycerol-alpha-phosphate (interacts with) malic acid | C |
| -0.7104 | < 0.0001 | 1 | interacts with | glutathione (interacts with) valine | C |
| 0.832 | < 0.0001 | 0 | interacts with | glutathione (interacts with) pyrophosphate | C |
| -0.7103 | < 0.0001 | 1 | interacts with | glutathione (interacts with) leucine | C |
| -0.7236 | < 0.0001 | 1 | interacts with | glutathione (interacts with) isoleucine | C |
| 0.7052 | < 0.0001 | 0 | interacts with | glucose-6-phosphate (interacts with) glycerol-alpha-phosphate | C |
| 0.7988 | < 0.0001 | 0 | interacts with | fumaric acid (interacts with) succinic acid | C |
| -0.7745 | < 0.0001 | 1 | interacts with | fumaric acid (interacts with) nicotinamide | C |
| 0.9549 | < 0.0001 | 0 | interacts with | fumaric acid (interacts with) malic acid | C |
| 0.7219 | < 0.0001 | 0 | interacts with | fumaric acid (interacts with) glycerol-alpha-phosphate | C |
| -0.7327 | < 0.0001 | 1 | interacts with | alpha-ketoglutarate (interacts with) isoleucine | C |
| 0.7534 | < 0.0001 | 0 | interacts with | alpha-ketoglutarate (interacts with) glutathione | C |
| 0.8827 | < 0.0001 | 0 | interacts with | alpha-ketoglutarate (interacts with) fumaric acid | C |
| -0.727 | < 0.0001 | 1 | interacts with | alpha-ketoglutarate (interacts with) valine | C |
| 0.8368 | < 0.0001 | 0 | interacts with | alpha-ketoglutarate (interacts with) succinic acid | C |
| 0.8925 | < 0.0001 | 0 | interacts with | alpha-ketoglutarate (interacts with) malic acid | C |
| 0.7462 | < 0.0001 | 0 | interacts with | fructose-6-phosphate (interacts with) glycerol-alpha-phosphate | C |
| 0.8265 | < 0.0001 | 0 | interacts with | fructose-6-phosphate (interacts with) glucose-6-phosphate | C |
| 0.9504 | < 0.0001 | 0 | interacts with | 3-phosphoglycerate (interacts with) phosphoenolpyruvate | C |
| 0.7724 | < 0.0001 | 0 | interacts with | palmitic acid (interacts with) stearic acid | C |
| -0.8024 | < 0.0001 | 1 | interacts with | pyrophosphate (interacts with) valine | C |
| 0.7176 | < 0.0001 | 0 | interacts with | phenylalanine (interacts with) valine | C |
